# Individual and Combined Cytotoxic Effects of Co-Occurring Fumonisin Family Mycotoxins on Porcine Intestinal Epithelial Cell

**DOI:** 10.3390/foods12132555

**Published:** 2023-06-30

**Authors:** Song Yu, Lianpeng Zou, Jiawei Zhao, Yiping Zhu

**Affiliations:** Division of Chemical Toxicity and Safety Assessment, Shanghai Municipal Center for Disease Control and Prevention, Shanghai 200336, China; zoulianpeng@scdc.sh.cn (L.Z.); zhaojiawei@scdc.sh.cn (J.Z.); zhuyiping@scdc.sh.cn (Y.Z.)

**Keywords:** fumonisins, gastrointestinal toxicity, combined toxicity, risk assessment, control strategy

## Abstract

Human health is seriously threatened by mycotoxin contamination, yet health risk assessments are typically based on just one mycotoxin, potentially excluding the additive or competitive interactions between co-occurring mycotoxins. In this investigation, we evaluated the individual or combined toxicological effects of three fumonisin-family B mycotoxins: fumonisin B1 (FB1), fumonisin B2 (FB2), and fumonisin B3 (FB3), by using porcine intestinal epithelial cells (IPEC). IPEC cells were exposed to various concentrations (2.5–40 μM) for 48 h, and a cell counting kit (CCK8) was used to determine cell vitality. Firstly, we discovered that they might inhibit cell viability. Additionally, the cytotoxicity of FB1 was significantly greater than that of FB2 and FB3. The results also indicated that the combinations of FB1-FB2, FB2-FB3, and FB1-FB2-FB3 showed synergistically toxicological effects at the ID10-ID50 levels and antagonistic effects at the ID75-ID90 levels. In addition, the FB1-FB3 exposure was also synergistic at the ID10-ID25 level. We also found that myriocin and resveratrol alleviated the cytotoxicity induced by fumonisin in IPEC cells. In all, this study may contribute to the determination of legal limits, the optimization of risk assessment for fumonisins in food and feed, and the development of new methods to alleviate fumonisin toxicity.

## 1. Introduction

Fusarium is the most influential pathogenic fungus affecting crops. Agricultural ear rot induced by means of Fusarium no longer solely leads to decreased yield. However, it additionally produces poisonous secondary metabolites, mycotoxins [1]. The World Food and Agriculture Organization (FAO) estimates that, every year, over 25% of cereal products are wasted globally due to mycotoxin infection [2]. Hepatotoxicity, nephrotoxicity, immunotoxicity, reproductive toxicity, and carcinogenicity of mycotoxins pose serious threats to human and animal health [3]. Fumonisins are toxic, low-molecular-weight, and water-soluble mycotoxins mainly produced by *Fusarium verticillioides* and *Fusarium proliferatum* [2]. Since they were discovered in 1988, 28 fumonisins have been recognized and categorized into the four most important groups: A, B, C, and P, which can be transformed to masked fumonisins by microbial or plant metabolism [4]. The main type of fumonisin contamination in nature was class B, including fumonisin B1 (FB1), fumonisin B2 (FB2), and fumonisin B3 (FB3). The most widespread or virulent type of contamination, and currently the main target of research, is FB1 [5,6].

Fumonisin contamination is widespread throughout the world in corn, wheat, rice, millet, oats, sorghum, soya beans, and relevant products [7]. An investigation indicated mycotoxin contamination exists in different regions of the world, including North America, Central Europe, Africa, South Asia, and Southeast Asia. Approximately 27% to 58% of crops were contaminated in these areas. The highest positive rate, 76%, was reported in South America (at a mean contamination concentration of 1.50 mg/kg) [8,9]. In 2012, FB1 and FB2 contamination rates in animal feeds in Korea were 50% and 40%, respectively [10]. From 2011 to 2013, the contamination rate of fumonisins (FB1 + FB2) in China’s Hebei province increased to 46.4% [11]. In 2014, the FB1, FB2, and FB3 contamination rate in maize products from Shandong province was 98.1%, with the highest levels being 5046, 1350, and 712.1 μg/kg, respectively, and 76.7% of maize samples were contaminated with FB1, FB2, and FB3 [12]. Our study found that the contamination rates of FB1, FB2, and FB3 in feed from 19 Chinese provinces were 93.15%, 91.78%, and 80.82%, respectively, with mean contamination levels of 150.82, 89.96, and 59.48 μg/kg [13]. A study showed that 40% of cattle feed was contaminated by fumonisin, with an average contamination level of 4.5 mg/kg [14]. Fumonisin can additionally contaminate different foods. Fumonisin has been detected in 37.5% of onion samples in the Taif place of Saudi Arabia [15]. Similarly, fumonisin contamination was found in 29% of raisin samples from the western vicinity of Greece, at levels ranging from 7.1–25.5 µg/kg [16]. Due to the thermal stability and corrosion resistance of fumonisins, it is difficult to eradicate contamination [9,17]. Once fumonisin enters human beings and animals, it endangers their health and causes huge economic losses [3,18].

It has been proved, in numerous studies, that FB1 involves multiple toxicities, including enterotoxicity [8]. Claudin and occludin-1 are the two dominant proteins in the tight junction of the intestine cells [19]. Mucins secreted through intestinal epithelial cells play a vital function in the intestinal chemical barrier, and mucins1 protects and lubricates the epithelial surface and mediates signal transduction [20]. FB1 has been claimed to disrupt the intestinal barrier towards these tight junction proteins [21]. Li et al. found that FB1 caused intestinal epithelial barrier impairment via endoplasmic reticulum stress induced by using the ceramide synthase two depletion [22]. mTOR-mediated autophagy also regulated FB1-induced intestinal inflammation using pyroptosis in vivo and in vitro [23]. FB1-induced intestinal injury with the aid of advertising intestine microbiota homeostasis was shown in another study [24]. Prenatal exposure to mycotoxins may lead to severe dysfunctions of the gut [25]. Exposure of FB1 and FB2 are risk factors for pregnant animals [26]. Although toxicity of FB2 and FB3 in cereals is not fully understood [27], FB2 and FB3 inhibit proliferation and induce cell apoptosis in gastric epithelial cells (GES-1) [9], and the negative effects of FB2 and FB3 cannot be ignored with regards to the potential of long-term exposure. Furthermore, as toxicology research has progressed, the synergistic toxicity effects of concurrent exposure to different toxins, such as FB1 and AFB1, DON and its derivatives, and AOH and AME, have been identified [28]. However, the combination toxicity effects of FB1 and other fumonisins have not been well investigated.

Generally, the digestive tract, as the first physiological barrier to foodborne toxin exposure, is the most vulnerable organ to toxins [29]. FB1 brings intestinal damage in both human and animals [30]. We suppose that FB2 or FB3 alone may also be able to induce enterotoxicity, and that synergistic or antagonistic toxic interactions may occur when the three fumonisins are present simultaneously. To meet the constraints of the 3R principle, the porcine intestinal epithelial cell (IPEC) is an in vitro model for fumonisin toxicity assessment in this study. The concentrations of FBs have been set at 2.5–40 μM, per the requirement of the fumonisin EU safety limit standard and common degrees of fumonisin in in vitro and in vivo research [8]. In the first step, we investigated the impact of fumonisins on cell viability. The combined toxic effects of fumonisin mixtures were subsequently evaluated and analyzed. Finally, the anti-fumonisin effects of myriocin (ISP-1) and resveratrol (RVT) were studied.

## 2. Materials and Methods

### 2.1. Chemicals

Fumonisin B1 (ab142433) and fumonisin B2 (ab142434) were obtained from Abcam (Cambridge, MA, USA). Fumonisin B3 (20434) was obtained from Cayman (Ann Arbor, MA, USA). Myriocin (476300-5MG) was obtained from Merck/Millipore (Billerica, MA, USA) [9]. A Cell Counting Kit-8 (CCK-8) (CK04-3000T) was purchased from DOJINDO Laboratories (Kumamoto, Japan). Antibiotic solution (streptomycin, penicillin, and amphotericin) (03-033-1B) and 0.25% trypsin solution (03-050-1B) were purchased from BioInd (Kibbutz Beit, Israel). Resveratrol (R107315) was purchased from Shanghai Aladdin Biochemical Technology (Shanghai, China). The fetal bovine serum (10099141C) was purchased from Invitrogen (Waltham, MA, USA). RPMI Medium 1640 (11835030) was purchased from Gibco (Waltham, MA, USA).

### 2.2. Cell Culture and Treatments

The Beijing Beina Chuanglian Biotechnology Institute (Beijing, China) provided the porcine intestinal epithelial cell line (IPEC) [8]. Penicillin-streptomycin-amphotericin B (BioInd) and 10% fetal bovine serum (Invitrogen) were added to RPMI medium 1640 (Gibco) in order to cultivate the IPEC cells. The IPEC cells in 6 cm dishes were treated with 1 mL 0.25% trypsin (EDTA) for 2 min, then added to 2 mL medium. The cells were harvested after centrifugation (1000 rpm, 5 min), and then they were passed according to a 1:2 ratio. The FBs (0–40 μM) and/or additional substances (15 μM RVT or 200 nM ISP-1) were added to the medium after they had attained 70–80% of their original volume. When fumonisin simultaneously treated cells, the concentrations of FB1, FB2, and FB3 were 3.125–25 μM, 1.875–15 μM, and 1.25–10 μM, respectively.

### 2.3. Cell Viability Assay 

In 96-well plates, 10,000 cells were added. They were then replaced with a culture medium containing varying amounts of fumonisin. Cell viability was then assessed using the Cell Counting Kit. Absorbance measurements were taken using a tablet reader known as the TecanGenios Pro.

### 2.4. Statistical Analysis

The data for this study consist of the mean and standard deviation of the three individual experiments. For one-way or two-way ANOVA, a Tukey or post hoc Bonferroni test were utilized after the event, and GraphPad Prism 8 (GraphPad Software Inc., San Diego, CA, USA) was used to assess the progressive differences between groups [9]. The probabilities were both two-sided and considered to be statistically significant up to 0.05 values. The Compusyn software program (ComboSyn Inc., Paramus, NJ, USA, http://www.combosyn.com (accessed on 12 November 2018)) was used to calculate all the parameters [9]. 

## 3. Results

### 3.1. Effect of Fumonisins on Cell Viability in IPEC Cells

**Figure 1:**
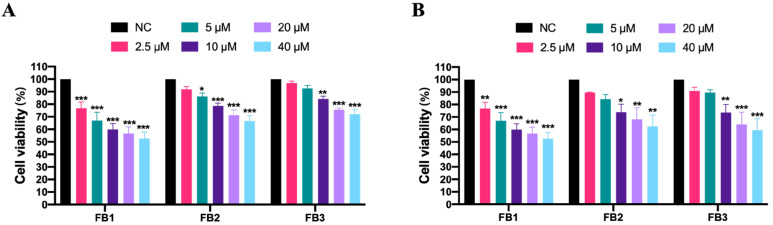
The cytotoxicity of fumonisins B (FBs) on the porcine intestinal epithelial cells (IPEC). IPEC cells were treated with FBs (0–40 μM) for 24 h (**A**) and 48 h (**B**) and then evaluated by the Cell Count Kit-8 cell proliferation assay. These numbers represented the three separate experiments’ mean ± SD (* *p* < 0.05, ** *p* < 0.01 and *** *p* < 0.001, analysis of variance (ANOVA) test). This NC represents the control group.

Cell viability is a commonly used measure of cytotoxicity. The CCK-8 kit was used in this study to detect FB cytotoxicity in IPEC cells. In the presence of 2.5–40 μM, cell survival rate decreased in a dose-dependent and time-dependent manner, as proven in Figure 1. After 48 h, the lowest level of cell survival rate was achieved in the 40 μM FBs group. The cell viability suppression rates were 63.01%, 37.57%, and 40.51% for FB1, FB2, and FB3, respectively. These findings indicate that FB1 was more toxic than FB2 or FB3. 

### 3.2. Combined Toxicity of Fumonisins in IPEC Cells

**Figure 2:**
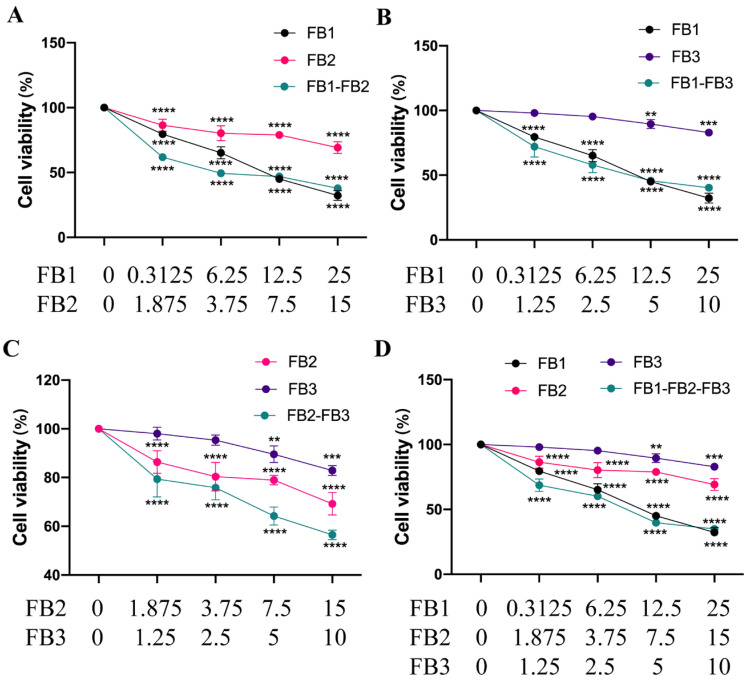
Interactions between FB1, FB2, and FB3, that affect the viability of IPEC cells. (**A**–**D**) Fumonisin was used to treat IPEC cells for 48 h either alone or in combination. These numbers represented the three separate experiments’ mean ± SD (** *p* < 0.01, *** *p* < 0.001, **** *p* < 0.0001, analysis of variance (ANOVA) test).

Figure 2 depicts the dose–effect relationship curve for the toxicity of the evaluated mixture as it relates to cell viability. The concentrations of FB1, FB2, and FB3 were 3.125–25, 1.875–15 μM, and 1.25–10 μM, respectively. The outcomes demonstrated noticeably lower cell viability in the binary or tertiary combinations. In each combination group, the cell viability decreased with increasing concentrations. The cell survival rate dropped to about 34.97% at a high dose of 25 μM FB1-15 μM FB2-10 μM FB3. In comparison to previous fumonisin mixes, the tertiary combination (FB1 + FB2 + FB3) was successful in lowering cell viability.

Table 1 displays the findings of the dose–response relationship parameters derived from in vitro cell viability investigations. The median-effect diagram provided the correlation coefficient (r). Given that there was a linear correlation coefficient, the data may be used for additional data analysis with the effect (inhibition of cell viability) equation. According to the isobologram method’s findings, the half-inhibitory dose (ID50) ranged between 9.37 and 37.26 μM in both the individual and combination treatment groups.

There are three essential sorts of interactions between toxin mixtures, particularly synergistic, additive, and antagonistic interactions. The kind of interplay between toxin combos can be assessed with the aid of calculating the combination index (CI) in accordance to the isobologram technique [31]. Table 2 showed the CI values of cytotoxicity (ID10-ID90) using the Compusyn software. The results show that the combinations of FB1-FB2, FB2-FB3, and FB1-FB2-FB3 were synergistically toxic to IPEC cells at the ID10-ID50 level. In addition, the combination of FB1-FB3 was also synergistic at the ID10-ID25 level. All combinations showed antagonistic effects at ID75-ID90 levels, especially for the combination of 25 μM FB1 and 15 μM FB2. The dose reduction index (DRI) indicated the dose reduction multiplier of the combined dose of the toxin under test compared to the dose of each toxin at the same rate of inhibition. The two DRI values, or at least one of them, are definitely opposite to the CI value in the numerically valued phrase. Table 3 also presented the results. When a synergistic effect occurs with a mixture, the value of the dose reduction index (DRI) favors dose reduction.

### 3.3. The Mitigation of FB-Induced Cytotoxicity by Natural Substances in IPEC Cells

**Figure 3:**
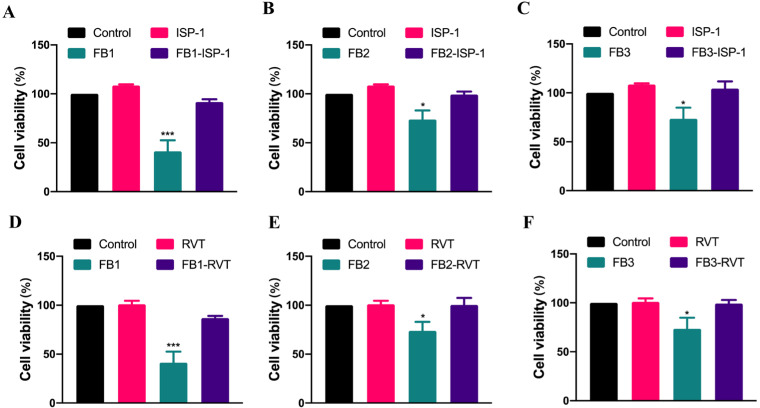
Myriocin (ISP-1) and resveratrol (RVT) alleviated FB-induced IPEC toxicity. (**A**–**C**) Effect of ISP-1 on cytotoxicity of FBs. (**D**–**F**) Effect of RVT on cytotoxicity of FBs. These numbers represented the three separate experiments’ mean ± SD (* *p* < 0.01 and *** *p* < 0.001, analysis of variance (ANOVA) test).

Furthermore, we explored novel strategies to mitigate the cytotoxicity of FBs. We evaluated the effects of two natural substances, myriocin (ISP-1) and resveratrol (RVT), on the induction of cytotoxicity by FBs. Our data showed that ISP-1 was able to reverse the inhibition of IPEC cell viability by FBs, with cell viability recovered from 41.31%, 73.86%, and 73.59% after FB1, FB2, and FB3 exposure to 91.60%, 99.31%, and 104.48%, respectively. Resveratrol showed similar results, with cell viability recovered to 86.84%, 100.36%, and 99.31% after the simultaneous exposures of resveratrol and FBs, respectively (Figure 3).

## 4. Discussion

Fumonisins extensively exist in various grain and feed products around the world [32]. In recent years, the toxicity and the impact of FB1 was gradually understood, with less research on FB2 and FB3 [27]. FB1 is associated with esophageal cancer and neural tube defects in human, as well as pulmonary edema in pigs, liver and kidney cancers in rodents, and horse leucoencephalomalacia [6]. Mycotoxins frequently cause harm to the intestinal system because they serve as the sites of both toxin exposure and accumulation [30]. Mycotoxins have been demonstrated, in numerous studies, to cause digestive tract damage [9,33,34]. Gunther et al. observed that fumonisin significantly reduced the villus height and crypt depth in the ileum, as well as the abundance of *Candidatus Savagella* and *Lactobaccilus* spp., and led to necrotic enteritis in broiler chickens [35]. For duodenal mucous, fumonisin could damage the duodenal mucous layer by inhibiting the expression of intestinal mucin 2 gene and changing the composition of mucin monosaccharides. In addition, it was able to reduce intestinal zinc transporter-1 gene expression and regulate intracellular methionine homeostasis in broiler chickens [36]. Vasileios et al. showed that fumonisins also inhibited the expression of antioxidant enzymes in intestinal cells and induced oxidative stress in broilers [37]. Martin et al. found that fumonisins not only damaged the intestinal barrier and microbial homeostasis, but they also reduced jejunal aminopeptidase N activity in weaned pigs [38]. FB1 was linked to enterotoxicity, but there was limited research on the enterotoxicity of FB2 and FB3 [30]. Currently, the development of safety standards for fumonisin relies on the risk assessment of single toxins to a great extent; however, the combined toxic effects between fumonisins are not fully understood, which may lead to inaccurate safety risk assessment [39]. Due to the progression of regulations on use of animals for experiments, cell-based systems are becoming more practical for assessing the effects of toxins or drugs. In this study, we utilized IPEC cells to evaluate the toxicity of fumonisins (FB1,FB2 and FB3), both alone and in combination.

It is initially required to evaluate the individual effect of each toxin to create a solid foundation for exploring their combined toxicity [40]. At first, the cytotoxicity of FBs was assessed in IPEC cells. In several investigations, cell viability was a crucial indicator to assess fumonisin toxicity. Our findings suggested that FBs largely suppressed cell proliferation. Regarding testing them as individual toxins, FB1 had a more significant effect compared to FB2 and FB3, while FB2 and FB3 were similar in their suppression. The highest hazard risk score was FB1, followed by FB2, and then FB3. Food and feed are frequently contaminated with FB1, FB2, and FB3 [41]. The intestinal combined toxicity of fumonisin was explored in the study. In all groups, FB1-FB2, FB2-FB3, and FB1-FB2-FB3 were shown to have a synergistic toxicity effect on IPEC cells at inhibitory concentrations of 10–50. In addition, the FB1-FB3 combination group was also synergistic at the inhibition concentration 10–25 level. All combination groups exhibited antagonistic effects at doses of inhibition concentration 75–90 level, in particular, the combination of 25 μM FB1 and 15 μM FB2. Prior research showed similar results [42,43]. At low concentrations, there was a synergistic toxicity for the combination of deoxynivalenol family mycotoxins. While, at high concentrations, it was an antagonistic effect [42]. Similar results were also seen when v79 cells were treated by citrinin and ochratoxin A [43]. However, compared with our previous results, the combination of fumonisin was prone to have a synergistic effect in GES-1 cells, but it had an antagonistic effect in IPEC cells [9,31]. In short, FB2 or FB3, as single agents, have mild toxicity. However, when mixing them with FB1, the impact on FB1’s toxicity should be considered.

The exposure of several toxins at once may have altered the single toxin’s initial processes of absorption, degradation, accumulation, and metabolism [44]. If the interaction of mycotoxin combinations is not now taken seriously in safety policies and restrictive standards, the risk may be underestimated or inflated, each of which could lead to economic losses. This is a new perspective that reflects the importance of considering synergistic or antagonistic outcomes of fumonisin combinations in future risk assessments.

FB1 can suppress ceramide synthase due to structural similarity with sphingolipid, which leads to a buildup of free sphingoid bases in cells [22]. Several studies suggest that this is one of the reasons for FB1-induced toxicity [45]. Moreover, FB1-inducing GES-1 cytotoxicity relies upon the disruption of sphingolipid metabolism [8]. The chemical structures of FBs consist of aminophenolic spines and a tricarboxylic acid group (-TCA), and it has been suggested that FB2 and FB3 may also have inhibitory effects on ceramide synthase in mouse liver [46]. Therefore, we investigated whether or not sphingolipid metabolism disturbance performs a role in the cytotoxicity of IPEC from FB2 and FB3. ISP-1, a potent inhibitor of serine palmitoyltransferase in the sphingolipid biosynthesis pathway, relieves accumulation of free sphingoid bases [47]. We found that the cell viability recovered to the same level as that of the control group when ISP-1 was given at 200 nM.

Applying natural active substances is considered as a safe method to prevent or alleviate the toxicity of food contaminants [48]. Numerous herbal plant extracts have been observed to alleviate the toxicity of mycotoxins [49]. The flavonoid luteolin attenuates injury of the intestinal epithelial barrier in Caco-2 cells [50]. Melatonin alleviates ochratoxin A-induced liver inflammation, involving intestinal microbiota homeostasis in a microbiota-independent manner in the previous study [51]. Resveratrol is an antioxidant with anti-inflammatory and anti-tumor properties. Recent research has demonstrated that resveratrol is capable on reversing the toxicity of mycotoxins [52], and it inhibits oxidative stress and apoptosis induced by aflatoxin B1 [53]. Yang et al. confirmed that resveratrol relieved the injury of intestinal epithelial cells triggered by the Nrf2 signaling pathway [54]. Our data showed that resveratrol with 15 μM effectively blocked the cytotoxicity of FBs in IPEC cells. In the future, animal experiments may be desired to explore effects of resveratrol in vivo.

## 5. Conclusions

IPEC cells were employed in this investigation as in vitro models to determine the toxicity of FB1, FB2, and FB3. We found that FBs notably decreased cell viability. We confirmed that the extents of toxicity were FB1 > FB2 > FB3. The interactions for the fumonisin combinations were synergistic or antagonistic effects, depending on concentrations of the toxins. Additionally, we found that myriocin (ISP-1) and resveratrol (RVT) likely reduced the cytotoxicity from FBs in IPEC cells. Finally, this outcome contributes to the determination of an acceptable range of fumonisin in the feed and food industry. Particularly, further attention and evaluations are needed on the synergistic effect of toxins at low concentrations. We additionally supply potential methods to manage FBs’ toxicities. 

## Figures and Tables

**Table 1:** Dose–effect relationship parameters for cytotoxicity by fumonisins in IPEC cells.

	Dm (μM)	M	r
FB1	11.3967	−1.0286	0.9968
FB2	28.9756	−0.4604	0.9699
FB3	37.2595	−1.1314	0.9955
FB1-FB2	8.0753	−0.4398	0.9719
FB1-FB3	11.5549	−0.6251	0.9795
FB2-FB3	24.1793	−0.5507	0.9859
FB1-FB2-FB3	9.3569	−0.7275	0.9756

Dm stands for dose the median-effect dose; M stands for the slope of median-effect curves; r stands for the correlation coefficient.

**Table 2:** Combination index (CI) for fumonisins’ cytotoxicity in IPEC cells.

Fumonisin	Joint Ratio	ID10	ID25	ID50	ID75	ID90
		Combination Index				
FB1-FB2	5:3	0.08	0.21	0.76	3.02	12.43
FB1-FB3	5:2	0.33	0.61	1.14	2.13	3.99
FB2-FB3	3:2	0.59	0.52	0.68	1.37	3.47
FB1-FB2-FB3	5:3:2	0.70	0.72	0.98	1.47	2.92

ID, inhibitory dose; CI < 1 signifies synergistic effects; CI = 1 signifies additive effects, and CI > 1 signifies antagonistic effects.

**Table 3:** Dose reduction index (DRI) for fumonisins’ cytotoxicity in IPEC cells.

Fumonisin	Joint Ratio	ID10	ID25	ID50	ID75	ID90
		Dose Reduction Index				
FB1	5:3	24.64	5.89	1.41	0.34	0.08
FB2		25.55	22.85	20.42	18.26	16.33
FB1	5:2	3.39	1.83	0.99	0.53	0.29
FB3		33.61	16.46	8.06	3.95	1.93
FB2	3:2	8.95	6.05	4.09	2.77	1.87
FB3		17.92	6.44	2.31	0.83	0.30
FB1	5:3:2	2.95	1.89	1.22	0.78	0.37
FB2		84.41	42.33	17.63	7.34	3.06
FB3		29.26	17.07	9.96	5.81	2.35

ID, inhibitory dose; DRI, dose reduction index.

## Data Availability

Data is contained within the article.

## Abbreviations

FBs	Fumonisin Bs
FB1	Fumonisin B1
FB2	Fumonisin B2
FB3	Fumonisin B3
CCK-8	Cell Counting Kit-8
DRI	Dose reduction index
GES-1	Human gastric epithelial cell line
IPEC	Porcine intestinal epithelial cells
ISP-1	Myriocin
ID	Inhibitory does
RVT	Resveratrol
